# Difficult intubation management in a child with I-cell disease

**DOI:** 10.4103/1658-354X.65121

**Published:** 2010

**Authors:** Abdul Kader M. Mahfouz, G. George, Suhaila S. Al-Bahlani, Mohamed Z. Al Nabhani

**Affiliations:** *Al Nahdha Hospital, Muscat, Sultanate of Oman*

**Keywords:** *Dental extraction*, *difficult intubation*, *I-cell disease*, *pediatric anesthesia*

## Abstract

I-cell disease (mucolipidosis II) is a rare metabolic disorder resulting from the deficiency of a specific lysosomal enzyme, N-acetylglucosamine-1-phosphotransferease. Developmental delay and growth failure are common presentations of I-cell disease. Psychomotor deterioration is rapid and progressive. Some physical signs such as hip dislocations, inguinal hernia, hepatomegaly, joint limitation, and skin changes may be present at birth. Coarse facial features and skeletal abnormalities become more conspicuous with time. The life expectancy of children with this condition is poor, with death usually occurring around the fifth year. A case report of the anesthetic management of gingivectomy with multiple dental extractions in a 5-year-old Omani female with I-cell disease is presented. The problems faced and their management during anesthesia are described.

## INTRODUCTION

I-cell disease (mucolipidosis II) was first described by Leroy and DeMars in 1967.[1] It is an autosomal recessive, metabolic storage disorder caused due to a deficiency of the enzyme N-acetyleglucosamine-1-phosphotransferase, which is one of two enzymes involved in the biosynthesis of mannose-6-phosphate.[2] This compound is a common marker enabling lysosomal enzymes to be transported to the lysosomal compartment of all cells. Without the transferase, lysosomal enzymes escape into the extracellular fluid resulting in marked elevation of lysosomal enzymes in plasma.[3] This transferase deficiency and consequent lysosomal depletion result in the accumulation of a number of macromolecules, mucopolysaccharides and mucolipids in the lysosome. This can be seen as coarse cytoplasmic granular inclusions in cultured skin fibroblasts giving rise to the name"I-cell" disease.[4]

The most common features of the condition are mental and physical retardation with typical orofacial features. Infants with I-cell disease are typically underweight at birth, with muscle hypotonia and coarse facial features, the full clinical picture of the disorder presenting between 6 and 8 months.[5] Other features include joint stiffness, dislocated hips, and tight, thickened skin, umbilical and or inguinal hernia, hepatomegaly, aortic insufficiency, and hazy corneas.[6] The atlanto-axial joint is unstable in I-cell disease due to an incompetent transverse ligament infiltrated by storage cells. A cartilaginous, rather than calcified, odontoid process may contribute to the instability.[7] The typical orofacial features in I-cell disease include: coarse facial features, high, narrow forehead, puffy eyelids, epicanthal folds, flat nasal bridge, anteverted nares, long philtrum, prominent gingival hyperplasia and macroglossia. Due to marked enlargement of the gingivae and alveolar process, the lower part of the face usually has a “fish-like” profile.[8] The gingival enlargement is progressive, from 4 months of age, to the extent that when jaw closure is attempted, contact is only made in the posterior part of the mouth, giving rise to an apparently open bite.

Diagnosis of the condition is often made in retrospect as a result of physical and mental delay. However, the presence of marked elevations of lysosomal enzymes in the plasma is an accurate diagnostic test for this disorder together with the presence of large lysosomal inclusions in peripheral lymphocytes.[3]

The life expectancy of children with I-cell disease is poor, as death occurs between the fifth and seventh year, from re current upper respiratory tract infections, bronchopneumonia, and heart failure.[9] For children with I-cell disease, the only therapeutic approach currently available is bone marrow transplantation to supply a source of structurally normal lysosomal enzymes.[10]

The anesthetic consideration in such cases included a high risk of difficult intubation and possibility required postoperative mechanical ventilation after the surgical procedure.

## CASE REPORT

**Figure 1:**
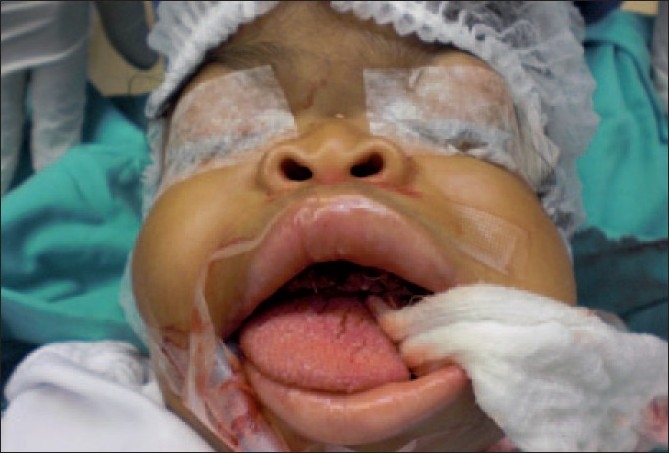
Patient at the end of surgery before extubation

An Omani female [Figure 1], 5 years and 11 months of age, was referred to Al Nahdha Hospital (tertiary Hospital for Oro-Maxillo-Facial services in Oman) from the Genetic Clinic where she was being followed-up regularly as a case of I-cell disease (mucolipidosis type II). She was the second of three siblings. One of them was well and free of the disease, whereas the other child died at the age of 1.5 years due to the same disease

She had the history of chronic nasal discharge and noisy breathing and also used to get recurrent chest infections for which she was admitted to hospital twice and treated with antibiotics and nebulization. On this occasion, she presented with generalized gingival hyperplasia, poor oral hygiene, and retained roots of lower anterior teeth. She was admitted for gingivectomy with multiple dental extractions under general anesthesia

On examination she was found to have: coarse facies, a depressed nose, a prominent forehead, high arched palate, widened wrists, a very large tongue, carious teeth, hypertrophied gums, generalized hypotonia, umbilical hernia, hepatomegaly, and thick skin. She weighed 7 kg and was noticed to have a developmental delay. Examination of the heart and lungs was unremarkable except for some conducted sounds over the lungs. Mallampati classification and range of neck movement could not be evaluated due to noncooperation of the child. Radiological examination revealed high arched palate with large adenoid and no shift of airway passages

### Anesthesia management

The child was premedicated with midazolam 3.5 mg orally and atropine 150 µg orally half an hour before the procedure. Due to the history of recurrent chest infection and the presence of conducted sounds over the chest, the child was nebulized with salbutamol solution 1 mg and ipratropium solution 75 µg half an hour before the procedure as a prophylactic measure against bronchospasm during anesthesia. The child was kept fasting for 6 hours before the procedure with a continuous IV infusion of dextrose 4.3% in 0.18 % normal saline at the rate of 28 mL/h.

On arrival in the operating theatre, routine monitoring was commenced which included electrocardiogram (ECG), non-invasive blood pressure (NIBP), pulse oximetry (S_PO2_) and end-tidal CO_2_tension (ET_CO2_).

General anesthesia was induced with sevoflurane 6% in oxygen. The patient's airway became progressively obstructed in spite of the use of an oro-pharyngeal airway size 1 and the jaw thrust manoeuvre. It was also not possible to assist her respiration with a bag and mask. A size 2 Laryngeal Mask Airway (LMA) was introduced and this successfully maintained the airway. Anesthesia was deepened subsequently. Due to the risk of bleeding from the planned surgery, endotracheal intubation was deemed necessary to guard against the risk of aspiration. After an adequate depth of anesthesia was reached, laryngoscopy and intubation were attempted after removing the LMA. Laryngoscopy was very difficult due to the stiffness of neck and it was not possible to properly position the patient for intubation. Because of this as well as the large tongue, the glottis could not be visualized (grade 4 according to Cormack and Lehane classification).[11] The LMA was reintroduced and another attempt of intubation was carried out with a Miller blade instead of the Macintosh blade, but it was still not possible to visualize the glottis. Once again the LMA was reinserted to maintain the airway. After instillation of vasoconstrictor nasal drops, intubation was attempted using a pediatric (3 mm) fibre-optic bronchoscope with a 4.5-mm uncuffed tracheal tube mounted on it. This had to be abandoned due to nasal bleeding from congested hypertrophied tissues and large adenoids, as well as rapid desaturation after removal of the LMA.

Finally, a sand bag was placed under the patient's shoulder to get some head extension and then the tip of epiglottis could be visualized (grade 3 according to Cormack and Lehane classification).[11] Oral intubation finally succeeded with a 4-mm ID endotracheal tube using a stylet, pushing the endotracheal tube behind the barely visualized epiglottis. Correct positioning of the tube was confirmed by auscultation of chest and by capnography. The tube was secured by adhesive tape and a throat pack was inserted. This manoeuvre was carefully used due to high risk of atlanto-axial dislocation in such type of patients.[7]

Anesthesia was maintained with sevoflurane in oxygen and nitrous oxide, with spontaneous respiration using a modified Jackson-Rees circuit. The respiration was assisted manually as required. At the end of the surgical procedure, the throat pack was removed and the pharynx cleared of secretions by suction. The patient was extubated when fully conscious and the recovery was uneventful.

## DISCUSSION

Anesthetizing a patient with I-cell disease requires skill, and it should only be undertaken by an experienced anesthetist. Many factors contribute to difficult intubation including large tongue, limited cervical movement and hypertrophy of nasal tissues, tonsils and adenoids due to the presence of inclusion bodies. As the children grow older, the airway becomes gradually more restricted as the initially swollen passages become stiff and easily traumatized.[12] Because of the poor growth potential, atrophied muscles, hypotonia, poor compliance of the narrow thoracic cage, and recurrent chest infection, the baseline respiratory status of these children is compromised and this may explain the need for postoperative mechanical ventilation in some cases.[13]

As general anesthesia is considered risky in this disease, it is recommended that elective surgical procedures be avoided as far as possible. If a procedure is considered essential, it should be undertaken at a major medical facility where pediatric anesthesia and pediatric critical care services are available. There should also be facilities to perform fibre-optic intubation. A detailed explanation of the anesthetic risk should be given to the parents.[14]

In the present case, the inability to maintain the airway without the use of a laryngeal mask airway limited the usefulness of the fibre-optic bronchoscope as a tool of intubation. In addition, bleeding occurred during a trial of fibre-optic intubation because of hypertrophied adenoids and congested nasal passage.

Although it was possible to maintain airway patency with a LMA, it was considered unsafe to perform the surgical procedure (gingivectomy and dental extraction) with a LMA *in situ* due to the risk of aspiration. FastTrack intubation using an intubating LMA was not possible in this case due to the unavailability of a suitable sized FastTrack LMA that matched the body weight of the child.

Many reports[12–13] have mentioned the possibility of difficult intubation as a risk factor during anesthesia, but in this case the inability to maintain the airway was an additional risk.
